# Medical Jurisprudence

**Published:** 1825-04-01

**Authors:** 


### MEDICAL JURISPRUDENCE.

1. *Culpable Abortion.** In the following case there are some facts of interest, and some points for criticism, notwithstanding the high rank which the reporter has attained in forensic medicine.

Messrs. Foderée and Ristelhueher having repaired to the house of the deceased, on the 7th March 1822, they found the body of Catherine S. extended on a table, and already exhibiting marks of incipient putrefaction, although death had only taken place the preceding day, after a short illness. On examination, there was nothing particular observable in the chest. In the abdomen, the peritoneum was found inflamed, and some spots of inflammation on the mucous membrane of the stomach and bowels. In the neighbourhood of the uterus there was a sanguineous effusion, with some clots of blood, in the midst of which was found a fœtus of about 60 days growth, with its umbilical cord. The womb was flattened, red, and inflamed. On more minute examination, it was found to be ruptured, the opening being about the size of a three franc piece. The internal surface of the uterus was also inflamed. The membranes

* Rapport sur une provocation d'Avortement par M. Foderée. Gazette de Santé, November, 1824.

of the fœtus were found perforated in two places—near the cervix uteri, and opposite the rupture in the parietes of the uterus, through which the fœtus had escaped into the abdomen. Nothing remarkable about the external parts of generation.

The medical commission called before them and examined several witnesses, by which it appeared that a midwife had been closeted with the deceased a day or two previous to her death, and had made use of a syringe with a long ivory pipe, which syringe was produced. Shortly after this interview, the deceased discharged a quantity of black blood from the vagina, and was seized with excruciating pains, which continued till death took place. From these data the Reporters came to the following conclusions:—viz. 1mo. That the deceased died of a violent inflammation with rupture of the uterus, followed by expulsion of the fœtus into the abdomen. 2ndo. That this inflammation and rupture being rare accidents, and not likely to take place, except as the effects or consequences of a grave malady, in the last stage of utero-gestation, it was probable that they were, in this instance, the result of violence, from the forcible introduction of the pipe of a syringe into the os uteri, and the injection of some acrid and stimulating liquid into the cavity of that organ.

The jury found the midwife guilty, and condemned her to ten years imprisonment.

Some strictures are made by the French Journalists on the testimony of Foderée. They observe that the medical man has no right to call or examine witnesses, in cases of this kind. He should confine himself to a rigorous examination of the body, aided by the light of physiology and pathology, leaving all matters of moral evidence to the investigation of the civil magistrate. This may be a good general rule; but we think there are many exceptions to it. For instance, in the above case, had no information of instrumental interference been obtained, there was nothing in the body which could have authorised the surgeon to pronounce upon such interference. The rupture of the uterus was not considered as resulting from the operation of the instrument, but from the contractile efforts of the organ itself—and the rupture of the membranes opposite the mouth of the womb might surely proceed from the same cause. It was, therefore, the moral evidence that decided the question. We read lately of a case of spontaneous rupture of the uterus, at an early period of utero-gestation, though we cannot recollect where. This early period, therefore, is no proof of criminal interference, though, backed by the moral evidence, it becomes a very strong presumptive one in the present instance.

The case is otherwise instructive as shewing the danger of procuring abortion by criminal interference.

